# Interventions for type 2 diabetes reduction among older people living with HIV in Harare

**DOI:** 10.4102/safp.v66i1.5827

**Published:** 2024-03-26

**Authors:** Nongiwe L. Mhlanga, Thinavhuyo R. Netangaheni

**Affiliations:** 1 Department of Health Studies, College of Human Sciences, University of South Africa, Pretoria, South Africa; 2 Department of Health Studies, Faculty of Human Sciences, University of South Africa, Pretoria, South Africa

**Keywords:** reduction, type 2 diabetes, older people living with HIV, interventions, Harare Urban District

## Abstract

**Background:**

Interventions for type 2 diabetes reduction among older people aged more than 50 years living with human immunodeficiency virus (HIV) are pertinent as they face excess risks amid a growing population of ageing people living with HIV (PLWH). This study aimed to describe interventions for type 2 diabetes reduction among older people living with HIV in Harare Urban District. The study was conducted in a low socioeconomic setting involving five primary health care clinics in Harare Urban District.

**Methods:**

A qualitative multi-method approach was applied using an exploratory descriptive design and an integrative review literature. The exploratory descriptive study collected data from two purposively selected samples: (1) older PLWH and (2) nurses. Whittemore and Knafl’s framework was used for the integrative literature review with articles from 2013 to 2023 selected. Data source triangulation was applied using Braun and Clark’s content analysis framework. Ethical approval was obtained (14056739_CREC_CHS_2022).

**Results:**

Twenty-three older PLWH with a mean age of 62 years, nine nurses with an average of 6 years’ experience, and 12 articles comprised the three data sources. The study found that screening should be from younger ages than the general population with assessment of HIV and antiretroviral (ART) specific risks and a diagnostic test for type 2 diabetes at ART initiation and routinely. Health education should provide information on adequate physical activity parameters and increased consumption of fruits and vegetables. Metformin may be considered as a pharmacological intervention where lifestyle interventions fail.

**Conclusion:**

The proposed interventions suggest measures to reduce type 2 diabetes and mitigate excess risks faced by older PLWH.

**Contribution:**

Improved screening, health education and pharmacological interventions for older PLWH in primary health care settings enable type 2 diabetes reduction.

## Introduction

The number of older people living with HIV (PLWH) is increasing. Globally, estimates from western countries show that at least 40% of PLWH are aged more than 50 years and this proportion of older PLWH is expected to increase to 73% by 2030.^1^ In Harare, which is located in the north east part of Zimbabwe in sub-Saharan Africa, it is estimated that by the end of 2035, the mean age of PLWH will increase to 45 years.^2^ With the increase in number of PLWH, there is increased likelihood of developing age-related chronic illnesses such as type 2 diabetes.^3^ In Harare, a twofold increase in type 2 diabetes is expected by 2035 where currently, prevalence is 6.9% among PLWH.^2,4^ For PLWH, the World Health Organization (WHO) denotes the chronological age of 50 years as indicative of older age, recognising that ageing occurs at least 10 years earlier than the general population.^5^ Therefore, in acknowledgement of the increased number of PLWH who are ageing and the associated likelihood of developing type 2 diabetes, healthcare worker-initiated preventative interventions for type 2 diabetes among older PLWH are critical.

Older PLWH face excess risk of type 2 diabetes because of common risk factors as well as HIV and antiretroviral therapy (ART) specific factors.^6^ Human immunodeficiency virus-specific risk factors include a long duration of HIV infection and the associated chronic inflammation, which leads to the development of type 2 diabetes.^6^ Antiretroviral therapy-specific risk factors include weight gain because of the use of older generation thymidine analogues such as stavudine and current integrase inhibitors such as dolutegravir.^7,8^ These excess risks have also been associated with the development of type 2 diabetes among PLWH from younger ages than the general population.^9^

Descriptions of excess risks associated with type 2 diabetes among older PLWH have also been coupled with recommendations to prevent some modifiable risks. The recommendations broadly described in extant literature from Canada, South Africa and Zimbabwe include screening, health education and pharmacological interventions^10,11,12^ Notably, these interventions are commonly used by healthcare workers for reducing type 2 diabetes among the general population. However, because of the excess risks and growing number of older PLWH, there is a need to improve these interventions.^11^ In improving these interventions, it is imperative to recognise best practices in high income countries, which are adequately resourced and make use of multidisciplinary teams.^13^

Concerning the screening for type 2 diabetes, there has not been an effective assessment tool available to screen older PLWH at risk of type 2 diabetes in several contexts.^14^ In a developed country such as the United States (US), current screening measures are inadequate, with 66% of PLWH tested for type 2 diabetes.^15^ Reasons for inadequate screening include underestimation of risks among older PLWH by health workers.^16^ Like in the US, in Zimbabwe, there are also no standardised tools for screening of non-communicable diseases (NCDs) among PLWH.^13^ Given this lack of guidelines in Zimbabwe, health workers do not screen for type 2 diabetes routinely among PLWH, with random blood glucose testing being performed for both PLWH and the general population indiscriminately to those who present with symptoms of type 2 diabetes.^12^ This lack of a standardised screening tool for type 2 diabetes among PLWH justified a study in Zambia, which assessed the practice of screening for signs and symptoms, and then conducting a random blood glucose test.^17^ The Zambian study, used a Chronic Health Care (CHC) checklist listing six signs and symptoms of type 2 diabetes followed by a random blood glucose test, if there was positive response to the six items.^17^ From this assessment, the CHC checklist failed to identify all cases of type 2 diabetes among older PLWH.^17^

In acknowledgement of the lack of standardised tools for type 2 diabetes screening and resultant inadequate screening, improved screening interventions among older PLWH have been recommended.^3^ In developed countries such as United Kingdom (UK) and Italy, such recommendations include screening for HIV and ART specific risks and routine diagnostic testing.^3,7^ Reiterating the recommendation for routine diagnostic testing, a narrative review concluded that testing should exclude the glycated haemoglobin (HbA1c) test as it underestimates type 2 diabetes among older PLWH.^18^ In Zimbabwe, the suggested interventions for screening from two studies recommended the integration of NCD and ART services.^12,18^ Notwithstanding the commendable evidence-based recommendations for screening, there is need for synthesis of this evidence to build on the current practices in Zimbabwe.^12,18^

Concerning health education, the promotion of physical activity and healthy eating habits is effective among the general population.^6^ Likewise, this education has also been provided to older PLWH with a mean age of 54 years in a study conducted in the UK.^6^ Despite the provision of health education on physical activity and healthy eating, a Canadian study found that older PLWH were aware of benefits of physical activity however, failed to meet requirements as they lacked knowledge on adequate parameters and faced other challenges like a lack of social support.^19^ On the other hand, in low-income countries in Africa such as in Tanzania, studies describing health education to reduce type 2 diabetes found that health workers ‘reminded’ PLWH to engage in physical activities.^20^ Moreover, a South African study recommended that health education should consider current physical activity and dietary habits of older PLWH.^11^ This consideration should acknowledge that older PLWH occupy themselves in several, varied forms of physical activity such as walking, domestic chores, aerobic exercise and work for economic purposes.^20^

Regarding nutrition education, a study in Dominican Republic found that during visits to ART clinics, both older PLWH and health workers valued receiving and providing nutrition education, respectively.^21^ Despite the value placed on nutrition education, a Zambian study concluded that older PLWH mostly consumed carbohydrates because of socioeconomic factors.^22^

Pharmacologic interventions for type 2 diabetes reduction have focussed on the use of the antidiabetic agent metformin. Findings on the use of metformin generally concur that metformin is effective in type 2 diabetes reduction; however, there are contrasting conclusions whether it should be used because of its adverse effects. In this respect, the Canadian narrative review concluded that metformin is effective in type 2 diabetes reduction, without any adverse effects.^23^ On the other hand, despite its effectiveness, the use of metformin should be used with caution for reduction of type 2 diabetes because of its interactions with some ART agents.^24^

Although there is consensus to enhance current screening, health education and pharmacological interventions currently implemented for the general public, there is a practical knowledge research gap where actual practice deviates from recommended practice.^13,18^ The actual practice is the utilisation of similar type 2 diabetes reduction interventions for both older PLWH and the general public, while older PLWH face excess risks to type 2 diabetes.^12^ The use of similar interventions for older PLWH and the general public is partly attributed to the evidence supporting improvements being fragmented and having been developed in various contexts. In light of the practical knowledge gap, there is a need to synthesise the evidence to improve current interventions. Therefore, the purpose of this study is to describe interventions for type 2 diabetes reduction among older PLWH in Harare Urban District.

## Research methods and design

The study formed part of a doctoral study at the University of South Africa, Department of Health Studies (project number Rec-240816-052). Two qualitative designs, namely an exploratory descriptive design and an integrative review of literature were used. Data source triangulation was then used to triangulate findings from the three data sources. Qualitative data triangulation refers to the use of various data sources to build a comprehensive understanding of a research problem.^25^ In data triangulation, focus is on a similar research question across the sources of data.^21^ In this study, three data sources were used: older PLWH, nurses providing care to older PLWH, and articles from an integrative review of literature. The main advantage of using data sources triangulation is that it improves the validity of results.^21^

The purpose of inclusion of older PLWH and nurses was to ensure that interventions developed are contextualised to Harare. The inclusion of older PLWH was justified because while using qualitative data to make recommendations, stakeholders who are targeted by the recommendations should be included.^26^ Moreover, inclusion of older PLWH minimises barriers to implementing interventions.^21^ The purpose of the integrative review was to provide a synthesis of evidence-based interventions, which could be wholistically built on the data from the local context, thereby minimising bias.^26^

### The exploratory descriptive study design

An exploratory descriptive study design was selected as it is most suitable for the development of healthcare interventions.^27^ The explorative descriptive study design was the first qualitative method and collected data from two sources: (1) older PLWH and (2) nurses.

The study setting of older PLWH and nurses was a low socioeconomic, densely populated, township community in Harare. Both populations were recruited purposively from ART clinics established solely for providing ART services. These were Glenview Polyclinic, Budiriro Polyclinic, Mufakose Polyclinic, Glenview Satellite Clinic, and Kambuzuma Polyclinic.

Therefore, the population that the researcher had access to was the accessible population, and the older PLWH were those accessing ART in the five clinics in Harare.

#### Inclusion criteria for older people living with HIV

Older PLWH aged more than 50 years willing to take part in the study and not diagnosed with type 2 diabetes were included in this study.

#### Exclusion criteria for older people living with HIV

Older PLWH unwilling to participate in the study, and older PLWH with mental illnesses without capacity to make a judgement on the risks and benefits of study participation were excluded from the study.

#### Inclusion criteria for nurses

Nurses in the ART clinics willing to participate were included in this study.

#### Exclusion criteria for nurses

Nurses not willing to participate in the study were not included in the study.

Purposive sampling was used to select both population groups (older PLWH and nurses providing care to older PLWH). Purposive sampling is a non-probability sampling technique based on the researchers’ judgement.^27^ For the older PLWH, judgement was based on physical appearance of age with confirmation of age on clinical records. The judgement used to select nurses was based on the researchers’ visual observation of the nurses’ providing services in the ART clinics after referral from the clinic managers and confirmation with the nurses that they provided ART services.

For both population groups, sample size was determined by data saturation, which refers to the point where further recruitment of participants does not yield new information.^27^

In-depth face-to-face interviews using two different semi-structured interview guides (one for older PLWH and the other for the nurses) were used for data collection. Data were collected in private rooms at the primary health care facilities with interviews conducted by the researchers. Data collection was performed from 05 December 2022 to 13 February 2023. The mean interview duration with older PLWH was 29 min, while the mean interview duration with nurses was 23 min. All interviews were audio recorded after participants consented. Interviews were conducted in Shona and English, with the Shona interviews being translated back-to-back by two Shona language experts during transcription. Transcription was carried out by the researcher N.L.M.

Data were analysed separately using Braun and Clark’s six steps of thematic content analysis for the older PLWH and nurses.^27^ The first step was data familiarisation that involved transcribing the data, reading and re-reading transcriptions.^27^ The second step was to categorise the data into codes; for the older PLWH the main categories that emerged were ‘economic activities as a means of physical activity and consumption of indigenous food like finger millet and pumpkin leaves’. Initial codes for nurses included ‘screening for risks’ and ‘encouraging consumption of indigenous food’. The second step also entailed checking that the codes generated relate to the research question. The third step was to organise the data into initial themes.^27^ The fourth step was to recheck the transcripts for additional data to support each theme. The fifth step was defining the themes, thus ensuring that themes align to the research question. The last step was data presentation.

The researchers considered how their professional backgrounds, experiences and personal assumptions could influence interactions with the participants. In this regard, the researcher T.R.N. was from an academic background, while the researcher N.L.M. was from a clinical background. As such, the researchers remained mindful of their research roles and maintained objectivity.

### Integrative review of literature

The second, qualitative method was utilised on the third qualitative data source was an integrative review of literature. An integrative review of literature is defined as a comprehensive systematic review of literature suited for supporting clinical decisions.^28^ Whittemore and Knafl’s updated five-step framework was used. The five steps are as follows: problem identification, literature search, data evaluation, data analysis and data presentation.^28^ The protocol for the integrative review of literature was registered with the Open Science Framework (registration ID: https://osf.io/8xumh).

### Problem identification

The first step was to identify the research problem.^28^ The Population, Intervention, Comparison and Outcomes (PICO) framework was used for problem identification.^29^ Table 1 shows the framework.

**TABLE 1:** The Population, Intervention, Comparison and Outcomes (PICO) framework.

Framework criteria	Problem identification
Population	The population included older PLWH aged more than 50 years.
Intervention	The interventions include descriptions of screening, health education, and pharmacological interventions for type 2 diabetes reduction among older PLWH.
Comparison	The comparison of interventions addressed: A comparison of different screening practices for type 2 diabetes reduction among older PLWH from various healthcare disciplines. A comparison of topics for health education. A comparison of use versus non-use of pharmacological interventions determined by adverse effects of drugs.
Outcomes	Outcomes for type 2 diabetes reduction among older PLWH to mitigate excess risks faced by older PLWH.

PLWH, people living with HIV.

### Literature search

The second step was to search the literature.^28^ This was performed by searching three databases, namely Cochrane Library, CINAHL and PubMed. The search was conducted using keywords: reduction, diabetes, people living with HIV. To search grey literature, targeted websites were searched. The literature search was conducted from 17 May 2023 to 02 June 2023 by two reviewers.

The inclusion criteria were:

- studies conducted between 2013 and 2023
- studies conducted in English
- studies that included PLWH aged 50 years or more.

The exclusion criteria were:

- studies not in English
- studies that did not include ‘older PLWH’, ‘ageing’ or ‘PLWH 50 years or more’.

### Data evaluation

To assess the quality of studies, the Critical Appraisal Skills Program (CASP) checklists were used which assessed the results, applicability, type and validity.^30^ Three types of CASP checklists were used: (1) cohort studies, (2) randomised control trials and (3) systematic reviews. Two reviewers assessed each article and consensus was reached through discussion. The evaluated studies were of high and medium quality.

### Data analysis

This step began with the extraction of demographic characteristics of the studies.^27^ The next step was an analysis of outcomes concerning type 2 diabetes reduction. The Noblit and Hare (1988) approach was used to build an interpretation.^27,31^ This involved reading and rereading the selected articles as a means of understanding study outcomes and recommended interventions.^27^ Studies with reciprocal outcomes were categorised.^27^ Synthesising similar study findings followed, for example, the screening category that included metaphors on excess risks. Lastly, a presentation of the synthesis was performed.^27^

#### Method for data triangulation

To triangulate the findings of the three data sources, Braun and Clark’s six steps of thematic analysis were reapplied. The themes from the three data sources were read and key similar codes were generated like varied physical activities. The codes were categorised into defined themes of screening, health education and pharmacological interventions. A review of the triangulated data was carried out.

### Ethical considerations

Ethical approval was granted by the College of Human Sciences Research Ethics Committee (reference no. 14056739_ CREC_CHS_2022). Permission to conduct the study was also granted by the City of Harare Health Department. Participants were provided with full information about the study and written consent was obtained from each participant. Means to ensure confidentiality included keeping all research information in a password-protected computer, only asking participants information about the study objective and respecting participants’ right not to share some information that they deemed secretive. Pseudonyms were also used for each participant to ensure anonymity.

## Results

Detailed results of physical activity and diet from older PLWH are outlined in an earlier study (Mhlanga & Netangaheni, 2024).^32^

### Characteristics of the older people living with HIV

A total of 23 older PLWH with an average age of 62 years participated. Most participants were female (52%). All the older PLWH stated that they commenced ART the same year they tested positive for HIV, meaning that average duration of HIV infection and ART use were similar; this was 11.3 years. Most (30%) older PLWH were retired.

#### Measures by older people living with HIV to reduce type 2 diabetes

Older PLWH shared their experiences of the physical activity and healthy eating habits they engaged in. Two main themes with five themes emerged.

**Main theme 1: Physical activity:** Older PLWH described the physical activities they engaged in which included working that enabled physical activity and following exercise routines. From this main theme, two themes emerged; working facilitates physical activity and older PLWH follow an exercise routine.

*Theme 1.1: Working facilitates physical activity*. The first theme that emerged was that working facilitates physical activity.This work would be in the form of economic activities, or domestic chores. Eight older PLWH described how their work facilitated physical activity. The older PLWH shared how economic activities such as farming, and vending were physically involving through heavy lifting and manual work. Domestic chores were also work that older PLWH described as physically involving and this included cleaning, cooking and gardening. The translated excerpts from Participants 11 and 8 who described how their occupation as a burial society coordinator, and domestic chores facilitated physical activity are as follows:

‘Normally I exercise through the work I do with my hands; I work in the garden. I ride my bicycle to and from Warren Park, so naturally that is my exercise that is my means of transport which helps me stay strong, I do it daily as I can be called to any burial society in Warren Park, to Kuwadzana I will be on my bicycle, I do not use a.’ (Participant 11, 63 years, male, burial society coordinator)

Participant 8 shared the nature of domestic work they were involved in that promoted physical activity. The translated quote is as follows:

‘I work in my house, I have no helper, I clean my dishes, I still can work with my upper body, I am only disabled waist downwards, I tend to my garden, I sweep outside I use two walking canes as I sweep my house, I do laundry.’ (Participant 8, 78 years, female, retired)

Moreover, while work promoted physical activity, the work older PLWH engaged in, facilitated routine walking. This is shown by a translated excerpt from Participant 20:

‘I go to work that’s how I exercise; I walk part of my journey to work, from town to Avondale, then I come back using a car, it is quite a distance to Lomagundi Street.’ (Participant 20, 56 years, female, office orderly)

The participants shared experiences of the physical work they did for economic purposes through their occupations, or domestic chores and concluded that working physically, which also promoted walking was a measure for a healthy lifestyle for type 2 diabetes reduction.

*Theme 1.2: Older people living with HIV follow an exercise routine.* Older PLWH shared that their physical activities included exercise routines. Three older PLWH described how they followed an exercise routine. These routines could be performed daily or weekly. Weekly exercise routines were described by participants who were mainly sedentary during the week, like Participant 3, an IT technician who would then exercise during the weekend and Participant 1 who was self-employed and ran twice a week. The translated quote from Participant 2 illustrates a daily exercise routine:

‘In the morning, I walk around my house, and a few hours later I exercise in the house by doing push-ups and before I sleep, I exercise by walking around the house again.’ (Participant 2, 62 years, male, no specified occupation)

From the older PLWH’s descriptions of weekly routines, which were performed at specific times and for specific durations it was concluded that participants followed exercise routines.

**Main theme 2: Healthy eating:** The second main theme was healthy eating. The older PLWH shared their experiences with healthy eating habits and two themes emerged. The first was healthy foods they consumed which included indigenous whole grains, fruit and vegetables. The second theme was that older PLWH’s diets were composed of mostly carbohydrates, and included proteins, fruits and vegetables in lesser proportions.

*Theme 2.1: Diet includes indigenous whole grains, fruit and vegetables.* Older PLWH shared that their diets included indigenous whole grains such as finger millet and sorghum as well as vegetables such as pumpkin leaves, amaranth leaves and blackjack. Four older PLWH described the consumption of indigenous whole grains, fruit and vegetables. Three of these older PLWH expressed that this food preference was influenced by growing up in a rural area. The following translated excerpt from Participant 6 illustrates this:

‘I grew up in rural areas we ate all types of Southern African Porridge, I grew up in Gutu where the finger millet Southern African porridge was the most common one which is what I eat most of the time now, in terms of vegetables I eat amaranth leaves, blackjack and okra we eat what we grew up eating.’ (Participant 6, 66 years, female, farmer)

Therefore, the older PLWH ate locally available whole grains, fruit and vegetables which was influenced by a rural background.

*Theme 2.2: Diet is mainly carbohydrate.* The last theme was that the diet of older PLWH was mostly carbohydrates. Nine older PLWH shared that their meal proportions were at least 50% carbohydrate with lesser proportions of vegetables, fruit and protein. Older PLWH also shared that they restricted the intake of sugar, salt and oils from their diets. The translated quote from Participant 17 is as follows:

‘I would give 50% to Southern African porridge, protein is 20%, vegetables are 30%; but sometimes vegetables are 20% and protein is 30%. I do not use a lot of oil in my food and with sugar it is just a few granules.’ (Participant 17, 73 years, male, retired, farmer)

As such the findings concluded that older PLWH consumed a diet mostly composed of carbohydrates.

### Results from nurses providing care to older people living with HIV

#### Sample characteristics

A total of nine nurses provided care to older PLWH participated. The mean age of the nurses was 42.8 years, with experience of working in ART clinics ranging from 2 to 12 years (mean 6 years). Most (78%) of the nurses who participated were female.

#### Interventions for type 2 diabetes reduction among older people living with HIV

The nurses providing care to older PLWH described the screening and health education interventions they performed for type 2 diabetes reduction. From the main theme of screening, three themes emerged. Concerning health education, two themes were generated.

**Main theme 1: Screening:** Nurses providing care to older PLWH shared that they screened older PLWH for type 2 diabetes and this screening included a risk assessment, an assessment of signs and symptoms, testing for hyperglycaemia and screening was supported by a multidisciplinary team. Seven of the nurses provided the description of screening for type 2 diabetes. The quote below summarises the screening measures undertaken:

‘Here in the OI clinic, we weigh our patients, those that are gaining excessive weight are the ones we target for testing, some we end up referring for further management.’ (Participant H, 54 years old, female, 11 years’ experience)

Therefore, from the participants’ descriptions, screening included other disciplines and risks like obesity were assessed.

*Theme 1.1: Screening for type 2 diabetes risks among older people living with HIV.* Nurses providing care to older PLWH shared that they screened older PLWH who are at risk of type 2 diabetes. The theme for screening for risks was drawn from responses from five nurses. In their description, Participant A noticed that such risks included weight gain. Moreover, Participant C observed the risk of family history of type 2 diabetes. The following quote supports this theme:

‘Here at the facility, we also do a risk assessment whereby we assess those at high risk of developing Diabetes, it is those with a family history of Diabetes.’ (Participant C, 33 years old, female, 4 years’ experience)

From the given quote, it was summed up that screening included a risk assessment, testing and referral to other disciplines.

*Theme 1.2: Screening for signs and symptoms.* Screening older PLWH for type 2 diabetes also included assessing those presenting with signs and symptoms of type 2 diabetes. Three participants described the screening for signs and symptoms. This was supported by the following quote:

‘You find that some of them are in the pre-diabetes stage, where we know if we manage them, we can prevent the onset of Diabetes, they have the symptoms so those we have to target for screening.’ (Participant D, 38 years old, 3 years’ experience)

From the shared experiences, nurses screened for signs and symptoms as a means of averting the development of type 2 diabetes among older PLWH.

*Theme 1.3: Screening includes random blood glucose testing.* Screening older PLWH also included random blood glucose testing; this was mainly done for older PLWH at risk or those presenting with signs and symptoms due to scarcity of resources. Three participants described the issue of random blood glucose testing. This is shown in the following quote:

‘We do not test everyone, the resources would not permit us, we only test those that present with symptoms of type 2 Diabetes, so we have to test those at risk because sometimes there are no strips.’ (Participant E, 40 years old, female, 2 years’ experience)

From the descriptions of screening those presenting with symptoms or those at risk, it was concluded that blood glucose screening was not conducted on all older PLWH because of resource limitations.

*Theme 1.4: Screening includes a multidisciplinary team.* The nurses providing care to older PLWH described the inclusion of laboratory personnel and medical doctors as part of the screening interventions. Two participants described the use of a multidisciplinary team. This is shown by the following quote by Participant D:

‘They come today and we record a high glucometer reading, then tomorrow we check them it is low, the next day it is high, it is fluctuating, those are the ones we target for referral to the lab, or when the doctor comes, they can see them.’ (Participant D, 38 years old, female, 3 years’ experience)

From the quotes, conclusion was made that screening older PLWH for type 2 diabetes included a multidisciplinary approach.

**Main theme 2: Health education:** The nurses also shared that they educated older PLWH on physical activity and healthy eating. All nurses interviewed described the health education on physical activity and healthy eating. The following quote illustrates the nature of health education provided:

‘Health education we have been providing on healthy eating. The exercising we also encourage it as well as is also part of the education.’ (Participant B, 41 years old, female, 6 years’ experience)

The above-mentioned quote summarizes the health education provided to older PLWH for type 2 diabetes reduction.

*Theme 2.1: Health education focusses on healthy eating and encourages physical activity.* Descriptions of the health education were provided. Health education was provided on the consumption of locally available food, and being physically active through domestic work, economic activities and walking. This description of healthy eating and physical activity was provided by all the nurses providing care to older PLWH. The quote supporting the health education is as follows:

‘We encourage them to eat unprocessed foods if they are eating Southern African porridge it should be from unrefined maize, and also to eat Southern African Porridge made from other grains like finger millet and to eat other whole grains like round nuts, the natural foods including wild fruits and seasonal fruits, whatever is available they should eat it and also traditional foods … we also encourage them to walk, we support them to be physically active because most of them they have small jobs they do in the home either in the fields or at their workplaces.’ (Participant H, 54 years old, female, 11 years’ experience)

The nurses’ interventions for type 2 diabetes reduction among older PLWH were focussed on health education and screening. The screening included the involvement of medical officers and laboratory personnel, a risk assessment, screening for signs and symptoms and random blood glucose testing. The health education promoted self-management measures of healthy eating and physical activity.

### Results from the integrative review of literature

**FIGURE 1:**
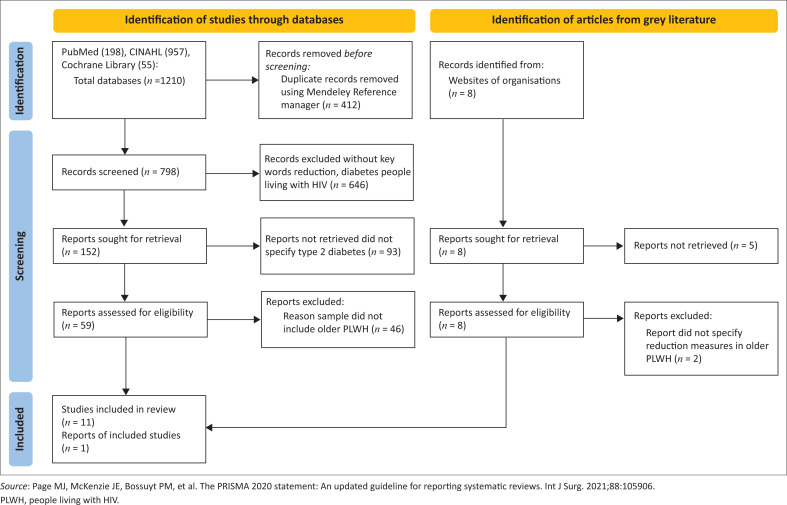
Preferred Reporting Items for Systematic Reviews and Meta-Analyses flow chart for integrative review of literature.

Studies screened from the three databases were 1210, and eight from the grey literature. We adapted the Preferred Reporting Items for Systematic Reviews and Meta-Analyses (PRISMA) flow chart illustrating the decision process as shown in Figure 1.^33^

#### Characteristics of the studies

From the 12 articles selected, one was a guideline, from the WHO. Eleven articles were studies. Concerning the geographical origins of selected research studies, two each were from the UK, Canada and Tanzania, one each was from Ghana, US, Saudi Arabia, Brazil, and Thailand. Five selected studies were from 2020, two were from 2023 and 2021 and one of each study was from 2013, 2019, and 2022. The studies also used different study designs, four used cross-sectional designs, and three were randomised control trials: systematic review, mixed method and longitudinal chart review. Table 2 lists selected studies’ characteristics.

**TABLE 2:** Characteristics of the articles reviewed.

Author and year	Geographical origin	Study design	Setting	Sample characteristics
Peters et al. (2013)	UK	Narrative review of literature	Not applicable	Focus on all PLWH; sample characteristics not applicable
Lazar et al. (2019)	US	Cross-sectional study	Participants recruited from New York City medical monitoring group	Most respondents older than 50 years; sample size of 397
Farahat et al. (2020)	Saudi Arabia	Retrospective longitudinal chart review	Participants recruited from the King Abdulaziz Medical City-Jeddah	Sample of 130 PLWH; mean age 50.1 years
Duncan et al. (2020)	UK	Mixed methods exploratory study	Participants recruited from three primary health care clinics in London	Sample size 23 PLWH; mean age 54 years
da Cunha et al. (2020)	Brazil	Cross-sectional descriptive quantitative survey	Participants recruited from an infectious diseases’ clinic at the Walter Cantídio University Hospital.	Sample size 125 PLWH; mean age 41.5 years
Isnard et al. (2020)	Canada	Randomised clinical trial	Participants recruited from Ottawa and Montreal	Sample size 23 PLWH; mean age 56 years
Nanditha et al. (2021)	Canada	Population based longitudinal cohort study	Medical records evaluated from British Columbia	Clinical records of 8031 PLWH and 32 124 HIV-negative people, median age 40 years
Sarfo et al. (2021)	Ghana	Cross-sectional comparative analytical study	Participants recruited from Kumasi recruited at Komfo Anokye Teaching Hospital	Adults more than 30 years; three cohorts: 258 on cART, 244 not on ART and 242 HIV-negative
Nimitphong et al. (2022)	Thailand	Randomised clinical trial	Participants recruited from an infectious diseases’ clinic at a teaching facility	Sample with mean age 49.6 years; sample size 74 with 37 in each cohort
Malindisa et al. (2023)	Tanzania	Cross-sectional study	Participants recruited from Mwanza district	Sample size 572 PLWH; mean age 43 years
Garrib et al. (2023)	Tanzania	Double-blind placebo-controlled trial	Participants recruited from four hospitals in Dar es Salaam; Amana, Mwananyamala, Temeke Hospital and Shree Hindu Mandal Hospital	364 participants; two trial arms, metformin and placebo; metformin trial arm median age 47 years and placebo trial arm 46 years
WHO guidelines on physical activity and sedentary life (2020)	Switzerland	Expert consensus and systematic review of evidence	Not Applicable	All population groups including older PLWH

*Source:* Please see the full reference list of the article for more information

PLWH, people living with HIV; ART, antiretroviral therapy; US, United States; UK, United Kingdom

The studies reviewed also had various study objectives and outcomes that related to the reduction of type 2 diabetes. Table 3 shows the study objectives and outcomes.

**TABLE 3:** Objectives and related outcomes of reviewed articles.

Theme	Study	Objective	Outcomes
Screening	Peters et al. (2013)	To describe screening for co-morbid conditions among PLWH	Screening should include diagnostic test on ART initiation and routinely; be from younger age; include ART and HIV risks and include routine diagnostic test
	Lazar et al. (2019)	To determine testing frequencies of comorbid conditions among PLWH and factors associated with testing	Screening should include ART-specific risks
	Da Cunha et al. (2020)	To assess prevalence and risks among people living with HIV	Screening should include ART exposure to adverse effects of ART
	Nanditha et al. (2021)	To compare prevalence and trends and age of onset of comorbidities among PLWH	Screening should commence at a younger age
	Sarfo et al. (2021)	To assess prevalence of prediabetes and overt type 2 diabetes among PLWH	Screening should commence at a younger age Screening should include diagnostic test
Health education	Farahat et al. (2020)	To assess prevalence and risks associated with type 2 diabetes	To provide health education on physical activity and diet among older PLWH
	Duncan et al. (2020)	To assess effectiveness and acceptability of a physical activity and diet modification for type 2 diabetes reduction	A modified STOP diabetes diet and walking 10 000 steps daily reduce type 2 diabetes
	Malindisa et al. (2023)	To assess dietary patterns and association with development of type 2 diabetes	A carbohydrate rich diet is associated with type 2 diabetes
	WHO guidelines on physical activity and sedentary behaviour (2020)	To provide recommendations for physical activity	Frequency, duration and intensity of physical activity among older people with chronic illness
Pharmacological interventions	Isnard et al. (2020)	To assess whether metformin can benefit PLWH	Metformin decreased weight in PLWH
	Nimitphong et al. (2022)	To assess efficacy of metformin in reducing diabetes among PLWH	Metformin reduced weight, HbA1c and insulin resistance among PLWH
	Garrib et al. (2023)	To determine the effect of metformin on PLWH with prediabetes	Metformin reduces blood glucose levels among PLWH

PLWH, people living with HIV; ART, antiretroviral therapy.

#### Interventions for type 2 diabetes reduction

The integrative review of literature synthesised evidence on three main themes: screening, health education and pharmacological interventions.

**Main theme 1: Screening:** The comparison of screening interventions among older PLWH for type 2 diabetes reduction yielded three themes. These included the following: screening should commence at a younger age, screening for ART and HIV specific risks, and screening should include a confirmatory laboratory test.

*Theme 1.1: Screening should commence at younger ages.* Three studies described the issue of screening for type 2 diabetes among PLWH from a younger age. The narrative review conducted from the UK recommended the screening for type 2 diabetes from the age of 40 years with consideration on the availability of resources.^34^ Concurring with the UK review, a Ghanian study found that prediabetes exists in PLWH from 44.9 years with overt type 2 diabetes by 50.6 years, and recommended that screening should commence at younger ages in PLWH.^35^ In a population-based cohort study that compared the onset of chronic conditions between PLWH and HIV negative people, PLWH developed type 2 diabetes at an earlier age than HIV negative people, with recommendation to screen for type 2 diabetes at an earlier age.^36^

*Theme 1.2: Screening for HIV and antiretroviral therapy induced excess risks.* The second theme was that older PLWH should be screened for HIV and ART induced excess risks. A study in the US which assessed the frequency of screening comorbid conditions among PLWH aged more than 50 years recommended the screening of ART-specific risks such as long-term adverse effects of ART.^15^ Screening for HIV and ART-specific risks was also recommended in a Brazilian study, especially among older PLWH who experience adverse drug effects from exposure to Stavudine and Zidovudine.^37^ Such screening for HIV and ART-specific risks should be done routinely and on ART initiation.^34^

*Theme 1.3: Screening for type 2 diabetes should include a routine diagnostic diabetes test.* Older PLWH screening for type 2 diabetes should include a routine diagnostic test, recommended by the WHO.^35^ The Ghanaian study recommended fasting glucose tolerance test, after finding a lower incidence (7%) type 2 diabetes among PLWH using HbA1c test, in comparison to a higher incidence (13.5%) using fasting glucose tolerance test.^35^ Similar recommendation is made by the UK review which highlights that fasting glucose testing should be done on ART initiation, and routinely every 6–12 months.^34^

**Main theme 2: Health education:** The second main theme that emerged from the integrative review of literature was health education for type 2 diabetes reduction among older PLWH. Studies, in different contexts, discussed the importance of providing health education on physical activity and diet.^6,38^ Complementing these studies, existent WHO guidelines on physical activity and sedentary behaviour 2020 stipulate physical activity parameters for older people living with chronic conditions like HIV.^39^

*Theme 2.1: Health education to promote adherence to a healthy diet.* To reduce type 2 diabetes among older PLWH, interventions should include health education on healthy eating. In a cross-sectional study from Tanzania, it was concluded that a diet that is not rich in vegetables among older PLWH is positively correlated with the development of type 2 diabetes.^40^ Among the older PLWH, older men consumed a carbohydrate-rich diet with lesser proportions of vegetables in comparison to older women^40^ The recommendation to provide health education on a vegetable-rich diet with less carbohydrates is validated in a mixed-methods exploratory study conducted in the UK among older PLWH. This study found that adherence to a diet consisting of at least seven servings of fruit and vegetables daily, with restrictions in carbohydrate to 600 kcal with 50% being whole grains, sugar to 25 mg and restrictions of oil to less than 10% of all total energy intake resulted in lowering of type 2 diabetes indicators.^6^

*Theme 2.2: Health education to promote physical activity.* Health education for type 2 diabetes reduction should also outline physical activities. The UK study found that walking at least 10 000 steps together with a healthy diet reduced the indicators of type 2 diabetes among older PLWH.^6^ In concurrence, the WHO 2020 guidelines on physical activity and sedentary life recommend 75 min - 150 min of vigorous physical activity or 150 min - 300 min moderate intensity physical activity or a combination of both for older people living with chronic illnesses. Regarding the nature of physical activity, all forms of physical activity including domestic physical activity, physical activity for economic purposes or leisure activities such as walking contribute to physical activity for older PLWH.^39^

**Theme 3: Pharmacological interventions:** The third theme described pharmacological interventions. Evidence to support interventions for type 2 diabetes reduction emanated from a study conducted in Canada, which used a sample of 23 PLWH with median age of 56 years and found that the use of metformin resulted in a weight loss of 1.4% of baseline weight after a 12-week trial.^41^ Similarly, with a larger sample size of 74 (37 in each cohort), a randomised clinical trial in Thailand found that metformin lowered the HbA1c and Homeostatic Model for Assessment for Insulin Resistance (HOMA- IR) levels in the metformin group, without adverse effects.^42^ Substantiating the effectiveness of metformin, in an African population, a randomised control trial with 364 respondents (182 in each group) in Tanzania found that metformin lowered fasting glucose levels among PLWH with a median age of 47 years.^43^ However, the study in Tanzania reveals that the difference in lowered fasting blood glucose levels between the metformin group and the control group was not significant and adverse drug events were reported among the metformin group.^43^

### Outcomes of the triangulation

The screening measures, health education or activities for physical activity and healthy eating and pharmacological interventions from the three data sources were synthesised. The findings of the triangulation are illustrated in Table 4.

**TABLE 4:** A Summary of data sources triangulation.

Main themes	Themes	Older PLWH	Nurses providing care to older PLWH	Integrative review of literature
Screening	Screening from a younger age	N/A	No	Yes
	HIV/ART-specific risk assessment	N/A	Yes	Yes
	Use of a multidisciplinary team	N/A	Yes	Yes
	Diagnostic testing	N/A	Yes	Yes
	Screening for signs and symptoms	N/A	Yes	No
Health education	Physical activity includes walking, domestic chores and economic activities	Yes	Yes	Yes
	Diet includes consumption of whole grains, vegetables, and fruit, with restricted oils, salt and sugar	Yes	Yes	Yes
	Diet includes a greater proportion of carbohydrates	Yes	N/A	No
Pharmacological interventions	Use of metformin	N/A	No	Yes

ART, antiretroviral therapy; PLWH, people living with HIV.

From the triangulation of the three data sources, interventions for type 2 diabetes reduction include screening from a younger age. HIV and ART-specific risks should be screened, which were described in an earlier study (Mhlanga & Netangaheni 2023).^44^ Screening should include a confirmatory laboratory test on ART initiation and routinely, which excludes the HbA1c test, and a multidisciplinary approach should be used.

Health education interventions should provide information on physical activity recognising that older PLWH perform activities like walking, domestic chores and economic activities, which are considered adequate should they meet WHO 2020 guidelines on physical activity. Nutrition education should foster consumption of whole grains, encourage increased consumption of fruits, vegetables with restricted intake of oils, sugar and salt. Regarding pharmacological interventions, metformin should be considered where lifestyle interventions fail.

## Discussion

The study found that screening could be improved by screening for excess risks, routinely and should include a confirmatory test excluding the HbA1c. Screening should involve a multidisciplinary team. Health education interventions could be improved by encouraging increased consumption of fruits and vegetables and adhering to WHO guidelines for physical activity. When diet and lifestyle interventions fail, the use of metformin can be considered.

Concerning the improvement of screening measures, previous studies in Zimbabwe concluded that screening and testing were not done routinely.^12^ Similarly, the lack of routine screening was highlighted in a study conducted in the US which found that 66% of PLWH are screened for type 2 diabetes.^15^ As such, interventions that foster routine screening described in this study demonstrate improvement in screening practices to identify all cases of type 2 diabetes. Regarding the screening for type 2 diabetes risks, the study in the US concluded that there is an underestimation of type 2 diabetes risks resulting in inadequate case finding of older PLWH at risk of type 2 diabetes.^16^ This study also affirms this underestimation of risks from the descriptions of the nurses, who highlight that interventions to reduce type 2 diabetes mainly target older PLWH who present with risks like weight gain, family history of diabetes, which are risks in the general population. However, the strength of the current study founded on the inclusion of the integrative review of literature, improves risk assessment, by including an assessment of HIV and ART specific risks like adverse effects of ART.^15,34,37^ The current study findings from the nurses also confirm the screening for type 2 diabetes signs and symptoms, then conducting a glucometer test which is a practice similar for the general population as described in another study conducted in Zimbabwe.^12^ This screening for signs and symptoms was also assessed in a Zambian study which concluded that case finding using this practice was inadequate.^17^ With the synthesis of evidence from the integrative literature review, testing could be improved by a routine diagnostic test with the exclusion of the HbA1c test.^34,35^

Health education could be improved by informing older PLWH on physical activity parameters that meet WHO parameters. Findings from nurse Participants, B and H, described ‘encouragement’ to exercise or walk, which validates findings from the Tanzanian study where older PLWH were ‘reminded’ to exercise.^45^ In addition, this study concluded that older PLWH engaged in physical activities like walking and domestic chores which confirms the findings in the Canadian study that concluded that older PLWH do engage in physical activity but may not be aware of adequate parameters.^19^ However, the current study findings were limited by inability to measure the intensity of physical activity performed by the older PLWH. As such, there is need for further study to ascertain the intensity of physical activity conducted and recommend the specific parameters which would require improvement among older PLWH. With regards to healthy eating, older PLWH described consumption of larger proportions of carbohydrates in comparison to fruits and vegetables and locally available whole grains. This finding confirms the study findings from the Zambian study which found that older PLWH consumed mostly carbohydrates.^22^ This consumption of a higher proportion of carbohydrates validates the need for nutrition education described in other studies from the UK and the Dominican Republic.^6,21^ The nurses also shared that they promoted consumption of whole grains, locally available fruits and vegetables, which confirms the healthcare worker prioritisation of nutrition education described in the Dominican Republic study.^21^ The improvement of nutrition education by fostering consumption of fruit and vegetables also illustrates the strength of the study collecting data from three sources. In this regard, data from older PLWH highlighted the gap in nutrition habits which was the source of nutrition education improvement.

The study also found that metformin could be used for type 2 diabetes reduction when lifestyle interventions fail. This finding was drawn from the integrative review of literature which drew randomised control trials on metformin effectiveness from three contexts: Thailand, Tanzania and Canada.^41,42,43^ Notably, these three studies were from 2022 and 2023, which implies a current global need for interventions to reduce type 2 diabetes among PLWH.

### Limitations

The study findings were limited by the small sample sizes in the explorative study design. The integrative review of literature was limited by different diagnostic tests to define the measures of type 2 diabetes among the reviewed articles; for example, two studies measured the effect of metformin in glycaemic control, one study assessed the effects using the HbA1c test, while another used fasting glucose testing.^37,38^

### Key recommendations

From these study findings, there is need for additional research that measures the intensity of physical activity currently performed by older PLWH. Such a study would identify the gap in adequacy of physical activity performed by older PLWH which could improve health education to reduce type 2 diabetes risks.

## Conclusion

The improvement of current type 2 diabetes reduction interventions is critical among older PLWH due to excess risks and an increasingly ageing population of PLWH. From the study findings, the proposed improvement should include screening for HIV and ART-specific risks, conducting a diagnostic diabetes testing on ART initiation and routinely, which excludes the HbA1c and screening from younger ages. Health education on physical activity and diet may be improved by consideration of older PLWH’s current physical activity routines with information on adequate parameters and encouragement on consumption of a vegetable-rich diet. These recommended interventions may contribute to the reduction of co-morbidities among older PLWH.
